# Laparoscopic hand-sewn cardioplasty: an alternative procedure for end-stage achalasia

**DOI:** 10.1007/s00423-021-02117-9

**Published:** 2021-03-24

**Authors:** Fátima Senra, Lalin Navaratne, Asunción Acosta-Mérida, Stuart Gould, Alberto Martínez-Isla

**Affiliations:** 1 General Surgery Department, London North West University Healthcare NHS Trust, Watford Road, Harrow, HA1 3UJ UK; 2 General Surgery Department, Hospital Universitario de Gran Canaria Doctor Negrín, Plaza Barranco de La Ballena, s/n, 35010 Las Palmas de Gran Canaria, Las Palmas Spain

**Keywords:** Cardioplasty, Laparoscopy, Achalasia, Lower oesophageal sphincter, Intraoperative endoscopy

## Abstract

**Background:**

Primary achalasia is a rare oesophageal motor disorder characterized by the absence of swallow-induced relaxation of the lower oesophageal sphincter and diminished or absent oesophageal body peristalsis. Around 5% of these patients will develop end-stage achalasia, where oesophagectomy may be advocated. We present the laparoscopic hand-sewn cardioplasty as an alternative ‘oesophagus-preserving’ procedure in patients with end-stage achalasia.

**Methods:**

We present a retrospective review of four patients who underwent laparoscopic hand-sewn cardioplasty. Data collected included pre-operative demographic information and investigations; and post-operative outcomes. Patients were scored pre- and post-operatively using Reflux Symptom Index, Eating Assessment Tool-10 and Voice Handicap Index-10 questionnaires.

**Results:**

Four patients underwent laparoscopic hand-sewn cardioplasty during the study period. In one patient, it was performed as a rescue procedure during attempted myotomy following multiple perforations of friable mucosa. In the other three patients, laparoscopic hand-sewn cardioplasty was performed for end-stage achalasia. None of the patients had post-operative complications and all patients were discharged on the second post-operative day. All patients experienced improvement in swallowing symptoms (EAT-10; *p* = 0.03) but developed post-operative gastroesophageal reflux.

**Conclusion:**

To our knowledge, this is the first published case series of laparoscopic hand-sewn cardioplasty for end-stage achalasia. It appears to be a safe and effective procedure for the treatment of end-stage achalasia, offering an alternative minimally invasive procedure to oesophagectomy. Laparoscopic hand-sewn cardioplasty can also be used as a ‘rescue’ procedure during myotomy in patients who have poor-quality mucosa which perforates intra-operatively or is at high risk of perforation/leaking post-operatively.

## Introduction

Primary achalasia is an uncommon oesophageal motor disorder of unknown aetiology characterized by the absence of swallow-induced relaxation of the lower oesophageal sphincter and diminished or absent oesophageal body peristalsis. This results in oesophageal stasis, progressive dilation and elongation of the oesophagus [1–3]. Current treatments aim to relieve obstruction at the lower oesophageal sphincter while minimizing the risk of post-procedure gastroesophageal reflux disease (GORD) [1, 3]. Heller’s myotomy combined with partial fundoplication is considered the gold standard surgical management for achalasia, with satisfactory results in the majority of patients [1]. However, disease progression occurs in some patients leading to end-stage achalasia, occasionally requiring oesophagectomy [3, 4]. In a recent systematic review and meta-analysis of 1307 patients who underwent oesophagectomy for end-stage achalasia, the pooled prevalence of pneumonia, anastomotic leakage and mortality were 10%, 7% and 2%, respectively [4]. We present the laparoscopic hand-sewn cardioplasty as an alternative ‘oesophagus-preserving’ procedure in patients with end-stage achalasia.

## Material and methods

### Patients

At our centre, 23 surgical procedures were performed for achalasia from January 2014 to December 2019. Of those, four patients underwent laparoscopic hand-sewn cardioplasty and were included for further analysis. All operations were performed by the senior surgeon (AMI). Data collected included demographic information, prior treatment for achalasia (e.g. myotomy), pre-operative investigations including pH studies and manometry, and post-operative outcomes. Post-operative outcomes included morbidity, length of post-operative hospital stay and symptomatic scoring at the sixth post-operative month. All patients were assessed with pre- and post-operative validated symptom index scales: Reflux Symptom Index (RSI) [6], Eating Assessment Tool-10 (EAT-10) [7] and Voice Handicap Index-10 (VHI-10) [8]. Quantitative data is presented as medians with range, and qualitative data as percentages. Statistical analysis of quantitative data was performed using a paired Student *T* test. A *p* value < 0.05 was considered statistically significant.

### Definition of end-stage achalasia

In the absence of therapy, or when therapy is inadequate, progressive dilatation and increasing tortuosity of the oesophagus can lead to end-stage disease in about 5% of patients [4]. End-stage achalasia was defined as the presence of a tortuous megaoesophagus (measuring 6 cm or more), and/or with recurrent obstructive symptoms after prior oesophageal dilatation or oesophagomyotomy [5]. The presence of end-stage achalasia was assessed by gastroscopy, barium swallow, 24-h pH monitoring and high-resolution manometry.

### Surgical technique: laparoscopic hand-sewn cardioplasty

**Fig. 1:**
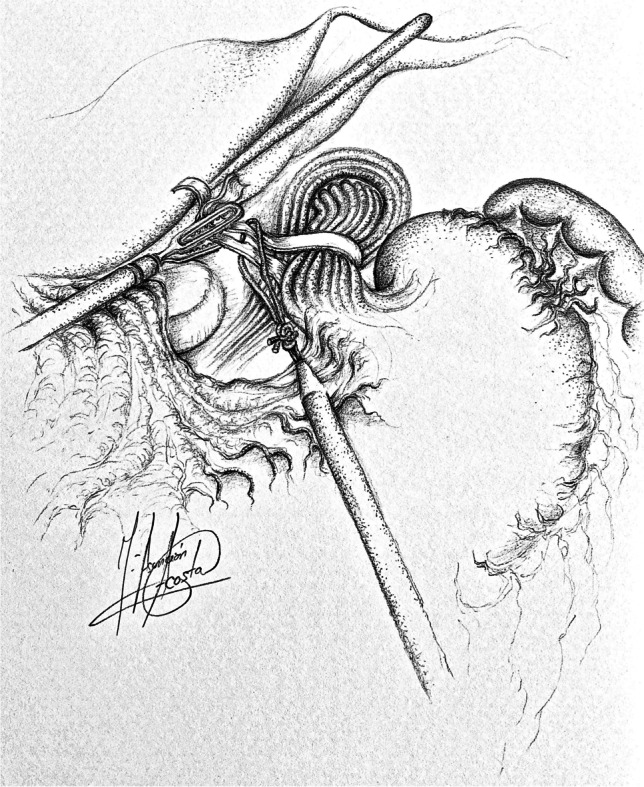
Dissection of the hiatus and tie surrounding oesophagus

**Fig. 2:**
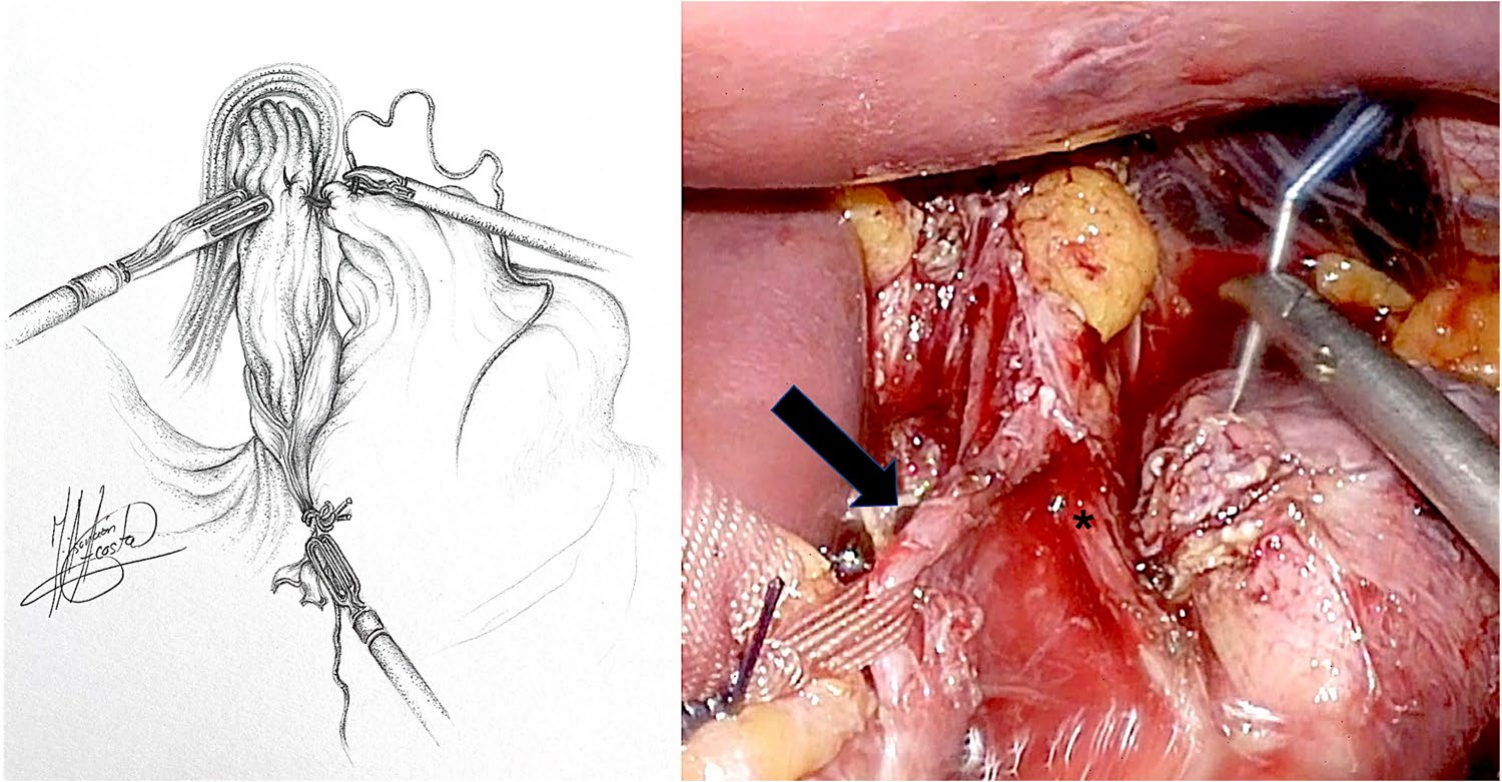
Preparation of anastomosis site: suture of the gastric fundus to the left side of the oesophagus

**Fig. 3:**
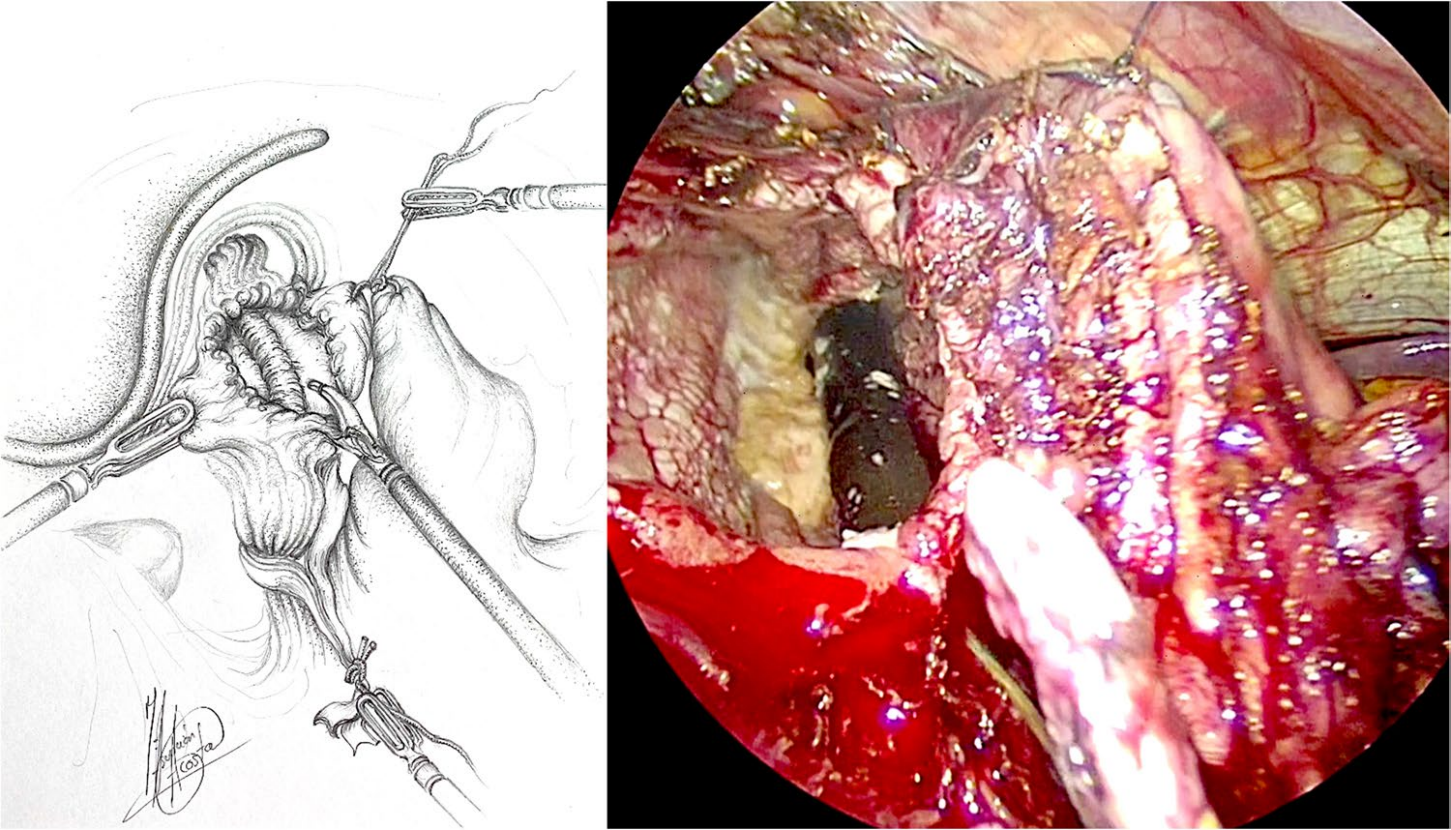
Oesophagotomy with endoscope inside

**Fig. 4:**
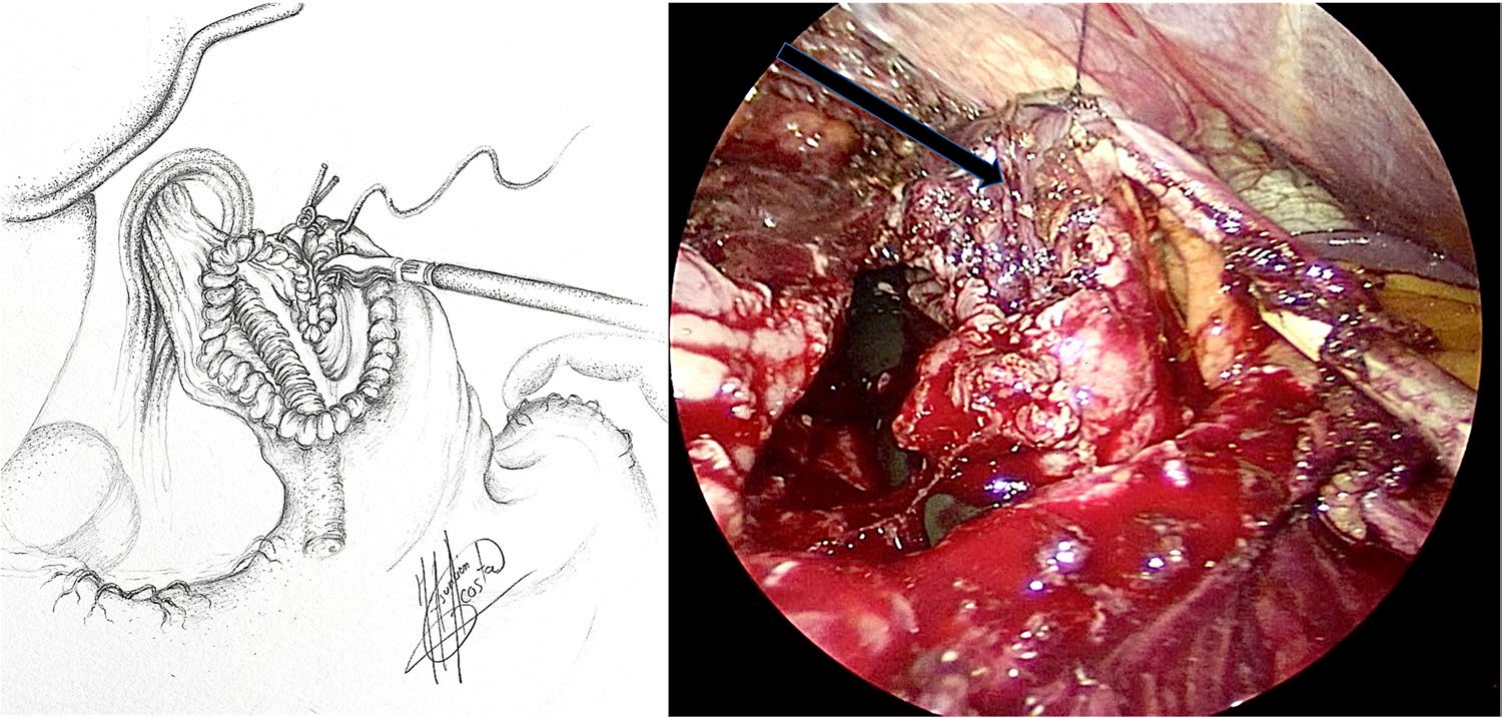
Gastrotomy and exposure of the angle of His. Suture of the posterior wall of the gastroesophageal anastomosis

**Fig. 5:**
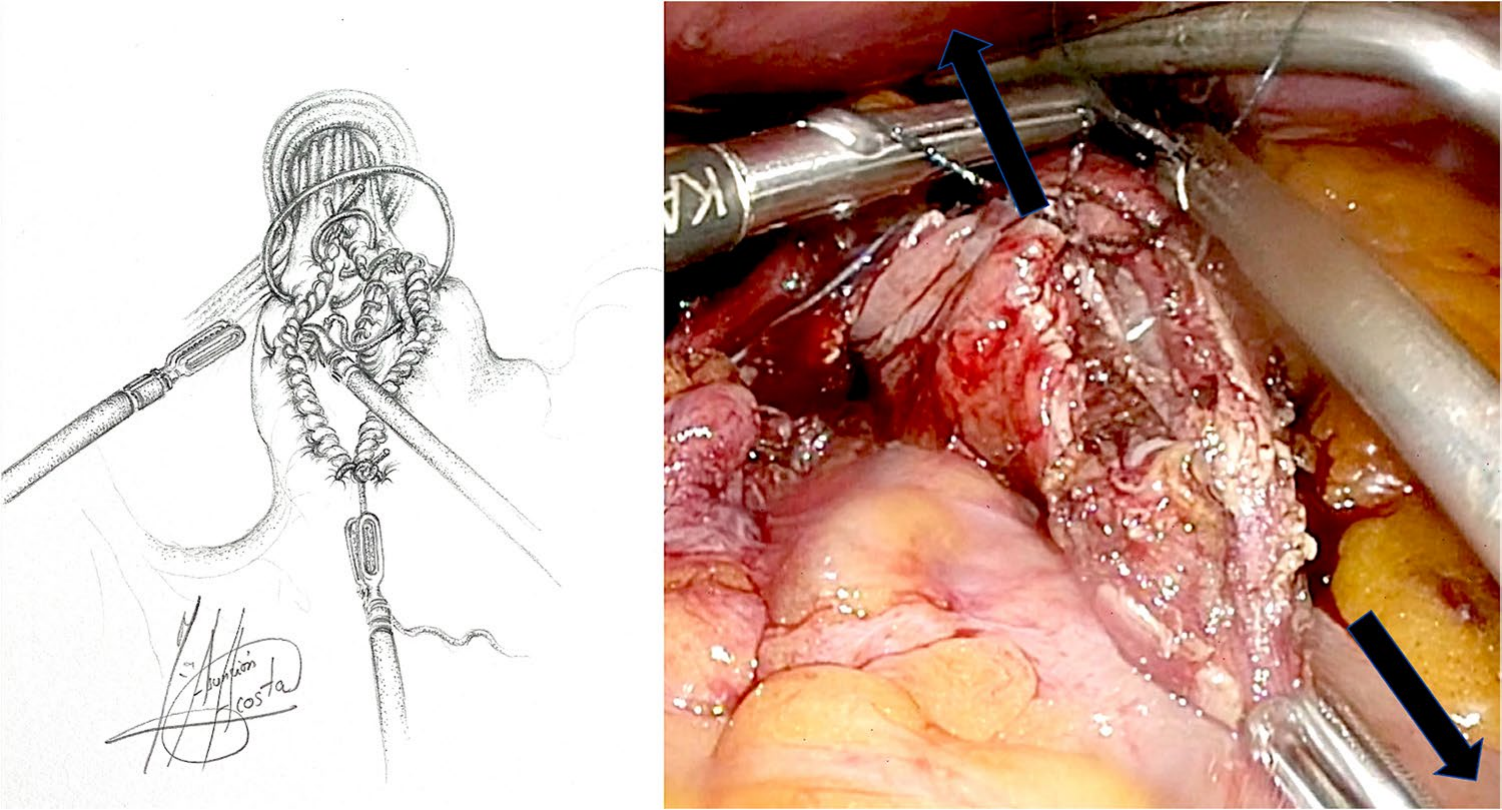
Suture of the anterior wall of the gastroesophageal anastomosis

Patients were formally consented in accordance with both local and international guidelines. All patients were placed on the operating table in the modified lithotomy position, in 20–45° of reverse Trendelenburg, with the surgeon standing between the patient’s legs and the assistant to the left side of the patient. A Nathanson liver retractor (Cook Medical, Dublin, Ireland) was inserted in the epigastrium to expose the oesophageal hiatus. A 5-m 30° laparoscope was inserted 10–12 cm below the xiphoid. Intra-operative gastroscopy was carried out in all cases not only to assess the oesophagus and locate the gastroesophageal junction, but also to fully evaluate the angle of His during construction of the cardioplasty. The hiatus was dissected, and if necessary, the oesophagus was encircled with nylon tape for further mobilization (Fig. 1). The anterior vagus nerve was identified, dissected and retracted laterally to the right using a nylon tape and an Endoloop® (Ethicon, New Brunswick, NJ, USA) to avoid iatrogenic injury during surgery (Fig. 2, arrow). The fully mobilized gastroesophageal junction was then retracted caudally and a stay suture was placed between the apex of the now free gastric fundus and the left side of the to prepare the gastroesophageal anastomosis site (Fig. 2, asterisk). With distal traction still being applied, an incision was made first in the oesophagus, with the flexible gastroscope inside (Fig. 3). If the patient had previously undergone myotomy (3 cases), the incision was performed over the previously exposed mucosa. Subsequently, a gastrotomy was performed at the apex of the fundus which was extended distally toward the oesophageal incision in a “U” shape allowing exposure of the angle of His and the gastroesophageal junction (Fig. 4). The gastroesophageal anastomosis was then performed with one or two layers of 3–0 V-Loc™ (Covidien, Mansfield, MA, USA) continuous barbed suture, starting with the posterior wall of the anastomosis (Fig. 4, arrow) and completed with the anterior layer (Fig. 5), facilitated with cephalad and caudal traction (Fig. 5, arrows). We did not perform a partial fundoplication in any of the patients to prevent post-operative GORD because it was not technically feasible in this operation. An intra-operative completion gastroscopy was always performed to assess the anastomosis, including an air-leak test, and to ensure that the endoscope could be passed through easily.

## Results

During the study period, four patients underwent laparoscopic hand-sewn cardioplasty for end-stage achalasia as an alternative to oesophagectomy. The median age of the patients was 39 years (range 33–56 years) and the male to female ratio was 3:1. Three patients who had end-stage achalasia had undergone previous laparoscopic Heller myotomy (LHM). Cases 1, 3 and 4 had Heller’s myotomy 4, 11 and 14 years respectively prior to undergoing cardioplasty. These patients were reviewed in the gastroenterology multi-disciplinary team (MDT) meeting, where peroral endoscopic myotomy (POEM) was considered an alternative intervention. However, the consensus decision was to perform a cardioplasty given the tortuosity of the oesophagus seen during pre-operative gastroscopy. In the remaining patient, laparoscopic myotomy was initially attempted, but due to multiple perforations secondary to friable, poor-quality mucosa, the index procedure was converted to laparoscopic hand-sewn cardioplasty. None of the patients underwent pre-operative endoscopic procedures. Pre-operative manometry was performed in all patients. Type II (Chicago classification) achalasia was diagnosed in one patient. In the remaining three patients, manometry could not be completed due to extreme oesophageal dilatation. All patient’s symptoms were pre-operatively assessed using the Reflux Symptom Index (RSI), Eating Assessment Tool-10 (EAT-10) and Voice Handicap Index-10 (VHI-10) questionnaires. Pre-operative median scores were RSI 25 (range 9–45), EAT-10 33 (range 21–40) and VHI-10 21.5 (range 0–28) (Table 1) [6–8]. This ‘triple’ assessment was used to assess patients pre- and post-operatively rather than using a single scoring tool (e.g. the Eckardt score [9]). This triple assessment allowed us to assess subjective scores for reflux and dysphagia, as well as voice symptoms.

**Table 1:** Pre-operative data

Case	Gender	Age (years)	Previous myotomy	Preoperative manometry (Chicago classification)	RSI	EAT-10	VHI-10
1	Male	54	Yes	Type II	9	30	18
2	Female	33	No	Not feasible	45	40	28
3	Male	41	Yes	Not feasible	39	36	25
4	Male	37	Yes	Not feasible	11	21	0

*RSI* Reflux Symptom Index, *EAT-10* Eating Assessment Tool-10, *VHI-10* Voice Handicap Index-10

All patients underwent a laparoscopic hand-sewn cardioplasty using the above described technique. All patients started oral feeding on the first post-operative day and were discharged on the second post-operative day. All patients were free from post-operative complication (Table 2).

**Table 2:** Post-operative data

Case	Hospital stay (days)	Morbidity	RSI	EAT-10	VHI-10	GORD requiring PPI
1	2	No	15	18	19	Yes
2	2	No	14	0	0	Yes
3	2	No	14	2	13	Yes
4	2	No	20	5	0	Yes

*RSI* Reflux Symptom Index, *EAT-10 *Eating Assessment Tool-10, *VHI-10* Voice Handicap Index-10, *GORD* gastroesophageal reflux disease, *PPI* proton pump inhibitors

All patients were re-assessed at the sixth post-operative month using RSI, EAT-10 and VHI-10 questionnaires. Post-operative median scores were RSI 14.5 (range 14–20), EAT-10 3.5 (range 0–19) and VHI-10 6.5 (range 0–19) (Table 2). There was significant improvement in swallowing symptoms following laparoscopic hand-sewn cardioplasty when post-operative EAT-10 scores were compared to pre-operative scores (*p* = 0.03). However, when comparing the pre- and post-operative scores of the RSI and VHI-10 questionnaires, no statistically significant differences were found (*p* = 0.39 and *p* = 0.25, respectively). All patients developed GORD, which was well controlled with lansoprazole 30–60 mg daily. None of the patients developed post-operative dysphagia and there have been no re-interventions required during within the median follow-up period of 32.5 (range 10–56) months.

## Discussion

This is the first published case series to our knowledge that reports the use of laparoscopic hand-sewn cardioplasty for end-stage achalasia, where the alternative proposed procedure would have been oesophagectomy. We describe four cases of laparoscopic hand-sewn cardioplasty with satisfactory outcomes in terms of safety and efficacy.

Extramucosal gastroesophageal myotomy, first described by Heller in 1913 and later modified by Zaaijer in 1923, achieves satisfactory long-term results in 85–95% of patients with achalasia [1, 10]. However, 10–15% of patients will experience deterioration of oesophageal function over time, and up to 5% will develop ‘end-stage’ achalasia (Rezende’s stage IV). End-stage achalasia results in a dilated and tortuous oesophagus that adopts a sigmoid shape, with sump formation near the hiatus [10, 11]. Failure of primary Heller’s myotomy may occur because of inadequate myotomy, recurrent adhesion/fibrosis after myotomy, incorrect or too tight fundoplication, fundoplication disruption, GORD, peptic stricture, end-stage achalasia and malignancy [2, 11, 12]. Among these causes, incomplete myotomy or recurrent adhesion/fibrosis after myotomy accounts for 50–60% of cases [11]. However, the underlying cause of failure cannot always be established with certainty [12]. Pre-operative investigations and intra-operative findings are usually unable to differentiate between inadequate myotomy and recurrent adhesion/fibrosis after myotomy [11]. Treatment options for failure of Heller’s myotomy include re-do cardiomyotomy, balloon dilatation of the gastroesophageal junction, peroral endoscopic myotomy (POEM) and, in severe cases, oesophagectomy [2, 10]. Oesophagectomy is indicated in patients with persistent or recurrent achalasia after failure of previous treatments and radiological progression of disease. However, oesophagectomy is associated with high morbidity and mortality rates (32.4% and 5.4%, respectively) [2]. Oesophagectomy is usually advocated in patients with a massively dilated oesophagus measuring more than 6 cm and/or with marked fibrosis, where dysphagia is unlikely to be resolved by dilatation or standard myotomy [2, 13].

Cardioplasty is a technique that has existed for over a century and has evolved over time. First described in 1913 by Heyrovsky and later modified in 1916 by Gröndahl, it has been used as an alternative to oesophagectomy in patients with end-stage achalasia [10, 14]. In 1972, Thal and Hatafuku described the creation of an esophagotomy that was further sutured to a patch of gastric fundus, creating a valve in the oesophagus, through a left thoracotomy for advanced achalasia (fundic patch operation) [15–17]. The technique was originally reported in 1964 for spontaneous rupture of the distal oesophagus [18]. In 2012, Dehn et al. published a modification of Gröndahl’s technique, creating a stapled oesophagogastrostomy through a gastrotomy using a laparoscopic approach, called laparoscopic stapled cardioplasty [12]. A full-thickness long myotomy should be done in the oesophagus, extending 5–6 cm into the oesophagus and 1–2 cm into the stomach to ensure full division of the lower oesophageal sphincter and destruction of the high-pressure zone [12, 14, 15, 19]. The length of the fundic gastrotomy should be proportional to the oesophagotomy [12]. The creation of an oesophago-gastrostomy allows appropriate oesophageal emptying even in the presence of poor motility. Patients presenting with poor oesophageal emptying with significant sump formation in the lower oesophagus, demonstrated on barium swallow, are good candidates for undergoing cardioplasty [10]. All patients should undergo pre-operative upper gastrointestinal endoscopy and barium swallow to assess the oesophagus and rule out carcinoma. Oesophageal manometry can be useful when endoscopy and barium swallow do not show significant changes, and the patient has developed recurrent dysphagia following previously successful treatment.

Three out of the four patients that underwent cardioplasty in our series had a previous Heller myotomy and suffered from end-stage achalasia. The remaining patient was undergoing primary laparoscopic myotomy, but due to poor-quality mucosa which perforated easily and compromised the safety of the myotomy, the procedure was converted to laparoscopic hand-sewn cardioplasty. Therefore, this procedure should also be considered a rescue operation during complicated primary or redo myotomy. The laparoscopic hand-sewn cardioplasty described here is similar to Gröndahl’s cardioplasty technique, but utilizes a minimally invasive approach. In one series, seven patients with recurrent achalasia following multiple failed medical treatments, including myotomies, were managed by laparoscopic stapled cardioplasty [12]. There were no anastomotic leaks reported and at 1 year, 86% of patients were free from dysphagia and 57% of patients were dependent on a proton pump inhibitor for GORD. The authors concluded that laparoscopic stapled cardioplasty may be a useful procedure for resistant achalasia. In another series, three patients underwent laparoscopic stapled cardioplasty for end-stage achalasia [11]. All three patients, who had each previously undergone one or two surgical myotomy procedures, achieved symptom improvement between the follow-up period of 5–24 months. The authors reported that the development of an angulated stapling device made this operation feasible using a laparoscopic approach. We advocate that a hand-sewn anastomosis is safe with low anastomotic leak rates and effective due to the creation of a wide passage through the cardioplasty. Moreover, the length of the anastomosis and the angle of His can be controlled more precisely under direct vision compared to a stapled anastomosis, where it is necessary to use intra-operative endoscopy to ensure the correct placement of the stapler. Occasionally, laparoscopic stapled cardioplasty can lead to incomplete division of the gastric and/or oesophageal mucosa, leaving mucosal bridges that can cause post-operative dysphagia [12]. This is a complication not seen with the laparoscopic hand-sewn cardioplasty as this stage of the procedure is performed under direct vision. Furthermore, problems related to mechanical staplers are also avoided with the hand-sewn technique. Laparoscopic cardioplasty with interrupted extracorporeal sutures has previously been described in one patient [20]. In this case report, a persistent 3-cm oesophageal stenosis despite an extended re-do oesophagogastromyotomy resulted in oesophageal stricturoplasty to avoid an oesophageal resection. The oesophageal mucosa was opened longitudinally for approximately 5 cm and the mucosal margins were sutured transversally with five extracorporeal interrupted sutures. By means of oesophageal stricturoplasty, this technique transposes the same principles of the Heineke-Mikulicz stricturoplasty for Crohn’s disease [21]. The authors reported that the procedure was free from complication, all pre-operative symptoms were resolved at 1-year follow-up and concluded that if confirmed by further cases, laparoscopic hand-sewn cardioplasty could become a valid option for a conservative treatment for these patients. Their technique was similar different to the one reported here, as we performed an intracorporeal two-layered closure with a continuous barbed suture which included an incision extending into the fundus of the stomach (by comparison our technique transposes the same principles of the Finney pyloroplasty). We believe that the lower morbidity of the procedure makes cardioplasty an appropriate surgical alternative in end-stage achalasia patients with very dilated oesophagus and ineffective oesophageal emptying that are not suitable candidates or willing to undergo oesophagectomy.

The creation of a wide gastroesophageal anastomosis in this operation offers post-operative rapid oesophageal emptying, improving symptoms related to achalasia, especially dysphagia. However, post-operative GORD with heartburn and regurgitation is common, which may develop over long term into stricture and/or Barrett’s oesophagus [10, 22–24]. In most series, including ours, these symptoms are well controlled with antacid medication such as proton pump inhibitors and overall improvement of symptoms is reported [10, 12]. In cases where GORD becomes intractable, a more extensive surgery should be considered, such as a Serrá-Dória procedure or oesophagectomy [12]. In 1961, Holt and Large suggested the use of a partial gastrectomy with Roux-en-Y reconstruction for the re-operation of achalasia with severe reflux oesophagitis secondary to a Gröndhal-type cardioplasty [21, 23]. In 1970, Serrá-Doria et al. combined a long latero-lateral gastroesophageal hand-sewn anastomosis partial gastrectomy and truncal vagotomy with a Roux-en-Y reconstruction in order to prevent acid and bile reflux for initial treatment of patients with Chagas megaoesophagus with good results [22, 25]. This technique has been used as an effective anti-reflux operation since 1985 with successful results [24, 26]. However, today, the main indication for cardioplasty with Roux-en-Y partial gastrectomy is for reoperation of achalasia due to reflux oesophagitis in high-risk patients that are not fit for oesophagectomy (old, malnourished patients or with concomitant cardiovascular or lung diseases), as it is associated to low morbidity and mortality [22, 24, 26].

The case series presented here has limitations. The small cohort population and the retrospective, non-random nature of this study are the primary limitations to generalizing the results. The other major limitation is the relatively short follow-up period of 6 months which evaluates short-term outcomes; however, long-term follow-up would be required to investigate late complications. Furthermore, due to the limited number of patients and follow-up, we are unable to comment whether or not the role of laparoscopic hand-sewn cardioplasty should be considered a ‘definitive’ or ‘bridging’ procedure for end-stage achalasia. For patients destined for oesophagectomy, this procedure may offer a bridge to allow for medical and/or nutritional optimization prior to definitive surgery. In addition, we are unable to provide a comparison of these results to a cohort of patients with end-stage achalasia who underwent oesophagectomy for end-stage achalasia. To date, previously published series, including ours, have reported safe and effective results in a small number of patients; however, laparoscopic cardioplasty is not commonly performed for end-stage achalasia. Prospective multi-centre studies are needed for patients with end-stage achalasia to identify which patients may benefit from this procedure and to compare the results of a stapled versus a hand-sewn technique.

## Conclusion

To our knowledge, this is the first published case series of laparoscopic hand-sewn cardioplasty for end-stage achalasia. From the limited data available, it appears to be a safe and effective procedure for the treatment of end-stage achalasia, offering an alternative minimally invasive procedure to oesophagectomy. Laparoscopic hand-sewn cardioplasty can also be used as a ‘rescue’ procedure during myotomy in patients who have poor-quality mucosa which perforates intra-operatively or is at high risk of perforation/leaking post-operatively.
